# Chronic IL-1-Exposed LNCaP Cells Evolve High Basal p62-KEAP1 Complex Accumulation and NRF2/KEAP1-Dependent and -Independent Hypersensitive Nutrient Deprivation Response

**DOI:** 10.3390/cells14030192

**Published:** 2025-01-28

**Authors:** Haley Dahl-Wilkie, Jessica Gomez, Anastasia Kelley, Kirti Manjit, Basir Mansoor, Preethi Kanumuri, Sammy Pardo, Dana Molleur, Rafah Falah, Anisha R. Konakalla, Morolake Omiyale, Susan Weintraub, Nikki A. Delk

**Affiliations:** 1 Biological Sciences Department, The University of Texas at Dallas, Richardson, TX 75080, USA; hcd160130@utdallas.edu (H.D.-W.); jessica.gomez2@utdallas.edu (J.G.); anastasia.kelley@utdallas.edu (A.K.); kirti.manjit@utdallas.edu (K.M.); basir.mansoor@utsouthwestern.edu (B.M.); pkanumur@sgu.edu (P.K.); rfalah@utdallas.edu (R.F.); anishareddy.konakalla@utdallas.edu (A.R.K.); morolake.omiyale@gmail.com (M.O.); 2 Department of Biochemistry & Structural Biology, The University of Texas Health Science Center at San Antonio, San Antonio, TX 78229, USA; pardo@uthscsa.edu (S.P.); molleur@uthscsa.edu (D.M.); weintraub@uthscsa.edu (S.W.)

**Keywords:** chronic IL-1, HMOX1, GCLC, p62-KEAP1, NRF2

## Abstract

Chronic inflammation is a cancer hallmark and chronic exposure to interleukin-1 (IL-1) transforms castration-sensitive prostate cancer (PCa) cells into more fit castration-insensitive PCa cells. p62 is a scaffold protein that protects cells from nutrient deprivation via autophagy and from cytotoxic reactive oxygen via NFκB and NRF2 antioxidant signaling. Herein, we report that the LNCaP PCa cell line acquires high basal accumulation of the p62-KEAP1 complex when chronically exposed to IL-1. p62 promotes non-canonical NRF2 antioxidant signaling by binding and sequestering KEAP1 to the autophagosome for degradation. But despite high basal p62-KEAP1 accumulation, only two of several NRF2-induced genes analyzed, *GCLC* and *HMOX1*, showed high basal mRNA levels, suggesting that the high basal p62-KEAP1 accumulation does not result in overall high basal NRF2 activity. Nutrient starvation induces NRF2-dependent *GCLC* upregulation and *HMOX1* repression, and we found that chronic IL-1-exposed LNCaP cells show hypersensitivity to serum starvation-induced *GCLC* and *HMOX1* regulation. Thus, chronic IL-1 exposure affects cell response to nutrient stress. While *HMOX1* expression remains NRF2/KEAP1-dependent in chronic IL-1-exposed LNCaP cells, *GCLC* expression is NRF2/KEAP1-independent. Furthermore, the high basal p62-KEAP1 complex accumulation is not required to regulate *GCLC* or *HMOX1* expression, suggesting cells chronically exposed to IL-1 evolve a novel NRF2-independent role for the p62/KEAP1 axis.

## 1. Introduction

Prostate cancer (PCa) is the second leading cancer type diagnosed in American men, with about 299,010 new PCa cases and over 35,250 PCa-related deaths estimated to occur in the United States in 2024 (America Cancer Society, cancer.org). The androgen receptor (AR) fuels PCa growth [1,2,3]; thus, PCa patients are treated with androgen deprivation therapy (ADT) and, upon recurrence, treated with anti-androgens to block AR activity [2,4,5,6]. While the 5-year survival rate for localized or regional PCa is nearly 100%, 10–20% of patients will inevitably develop lethal castration-resistant PCa (CRPC) due to promiscuous or overactive AR activity in PCa cells or, conversely, due to PCa cell adaptation to low or no AR accumulation [5,6,7,8].

Chronic inflammation is implicated in cancer initiation and progression, and the tumor microenvironment is replete with inflammatory cytokines and chemokines secreted by tumor cells and immune cell infiltrates [9,10,11]. We and others have shown that acute (i.e., days) treatment with the inflammatory cytokine, interleukin-1 (IL-1), is sufficient to represses *AR* and AR target gene expression in PCa cells while concomitantly upregulating other prosurvival proteins and pathways [12,13,14,15,16]. Thus, acute IL-1 exposure can, de novo, select for viable AR negative/low PCa cells that would be insensitive to ADT or anti-androgens. Because chronic inflammation drives cancer progression [11,17] and IL-1 is elevated in patient serum, is associated with poor PCa prognosis and a high Gleason score, and promotes PCa metastasis [18,19,20], we sought to determine if chronic IL-1 exposure would also select for viable AR negative/low PCa cells. Using LNCaP [21] and MDAPCa2b [22] cells, we discovered that chronic exposure (i.e., months) to IL-1 selects for cells that evolve insensitivity to exogenous IL-1 and, thus, the cells restore their AR levels and activity. However, LNCaP cells chronically exposed to IL-1 acquire AR independence [21]. We previously reported that sequestome-1 (hereinafter, p62) is upregulated by acute IL-1 concomitant with *AR* repression [13,15] and that p62 is cytoprotective for AR-independent PCa cell lines [23]. Therefore, we sought to determine the regulation and function of p62 in LNCaP cells that have been chronically exposed to IL-1 and have acquired AR independence.

p62 is a scaffold protein that, to date, has been shown to interact with over a dozen different proteins, implicating p62 in multiple biological and tumor-promoting processes, such as motor protein trafficking [24], cell proliferation [25], tumor initiation and growth [26], and metastasis [27]. The most well-characterized p62 roles are in autophagy and antioxidant response [12]. Cancer cells have high basal autophagy to maintain cellular homeostasis and to meet high metabolic demands [28], and cytotoxic reactive oxygen species (ROS) are abundant in the inflammatory tumor microenvironment [29], forcing tumor cells to mount cytoprotective antioxidant responses. p62 helps maintain cellular health and nutrient availability by sequestering cytotoxic protein aggregates, damaged organelles, and microbes into the autophagosome for degradation and biomolecule recycling by binding the autophagosome membrane protein, microtubule associated protein 1 light chain 3 (LC3) [12,30,31,32,33,34,35]. To attenuate IL-1-induced ROS, p62 binds and poly-ubiquitinates tumor necrosis factor receptor-associated factor 6 (TRAF6), leading to the downstream activation of the antioxidant transcription factor, nuclear factor kappa light chain enhancer of activated B cells (NFκB) [36,37]. Additionally, in response to ROS, p62 competitively binds Kelch-like ECH-associated protein 1 (KEAP1) to sequester KEAP1 from nuclear factor (erythroid-derived 2)-like 2 (NRF2), enabling NRF2 to translocate to the nucleus to induce antioxidant genes [38,39,40]. Finally, NFκB [41,42] and NRF2 [12,43] induce *p62* expression in an ROS-dependent manner to attenuate reactive oxygen stress. Thus, p62 functions in a positive feedback loop to mediate ROS activation of NRF2 [12] and IL-1 activation of NFκB [41]. p62 has many diverse functions, but the role and mechanism(s) of p62’s function in CRPC and anti-androgen resistance has not been fully explored, despite its established central role in cancer. We have shown that p62 is cytoprotective for AR-negative, CRPC cell lines, PC3, and DU145 [15,23,44], and another group recently demonstrated the p62/KEAP1 axis promotes NRF2-dependent PCa cell proliferation, survival, and invasion in DU145 cells [45]. To explore the mechanistic regulation and function of p62 in CRPC development, in this study, we start by investigating p62 protein interactions using our novel chronic IL-1-exposed LNCaP sublines.

## 2. Materials and Methods

### 2.1. Cell Culture

The LNCaP (ATCC, Manassas, VA, USA; CRL-1740) PCa cell line was maintained in a 37 °C, 5.0% (*v*/*v*) CO_2_ growth chamber, cultured in Dulbecco modified eagle medium (DMEM (Gibco/Thermo Scientific; Waltham, MA, USA, USA;1185-092) supplemented with 10% (*v*/*v*) fetal bovine essence (FB essence (FBE); Seradigm, Radnor, PA, USA; 3100-500), 0.4 mM L-glutamine (L-glut; Gibco/Invitrogen, Waltham, MA, USA; 25030-081), and 10 U/mL penicillin G sodium and 10 mg/mL streptomycin sulfate (pen-strep; Gibco/Invitrogen, Waltham, MA, USA; 15140-122). The chronic IL-1 sublines were generated and maintained as described in Dahl et al. (2020) [21]. Cell line authentication for LNCaP parental cell lines (-1,-2,-3) and chronic IL-1 sublines (LNas1,-2,-3 and LNbs1,-2,-3) was performed by STR profiling by the DNA Genotyping Core, UTSW Medical Center. The LNCaP cell line used in this study is commercially available and was purchased from the American Tissue Culture Collection (ATCC). The three sets of chronic IL-1 sublines were derived in our lab from the LNCaP cell line as described in [21], and their reproducible phenotype is confirmed (Figure S1). We hereby confirm that none of the cell lines used require any ethics approval for their use.

### 2.2. Cell Treatments

Cytokines: IL-1α and IL-1β cytokine treatments were prepared as previously described in [21]. Gene silencing (siRNA): The following siRNA concentrations were used: 70 nM non-targeting siRNA (Dharmacon, Lafayette, CO, USA; D-001206-13-05), *p62* siRNA (Dharmacon, Lafayette, CO, USA; M-003400-02-0005, pool of 4 oligos), *KEAP1* siRNA (Dharmacon, Lafayette, CO, USA; M-012453-00-0005), or *NRF2* siRNA (Dharmacon, Lafayette, CO, USA; M-003755-02-0005). Cells were transfected with siRNA using siTran 1.0 transfection reagent (Origene, Rockville, MD, USA; TT300003) DMEM/2.5% FBE growth medium for 4 days. Serum starvation: Cells were plated in DMEM/2.5% FBE growth medium, and once the cells were attached and semi-confluent, the medium was replaced with DMEM/0% FBE (serum starvation) or DMEM/10% FBE (replete medium control).

### 2.3. RNA Isolation and Reverse Transcription Quantitative PCR (RT-qPCR)

Total RNA was extracted, reverse transcribed, and analyzed by RT-qPCR as previously described [15]. Primer sequences for genes of interest are listed below (MilliporeSigma, Burlington, MA, USA). Gene of interest cycle times (CT) were normalized to the β-actin. Relative mRNA levels were calculated using the 2^−ΔΔCT^ method. Primer sequences, 5′-3′: *Sequestome-1 (p62)*, forward AAATGGGTCCACCAGGAAACTGGA, reverse TCAACTTCAATGCCCAGAGGGCTA; *Beta actin (β-actin)*, forward GATGAGATTGGCATGGCT TT, reverse CACCTTCACCGGTCCAGTTT; *NAD(P)H dehydrogenase (quinone) (NQO1)*, forward GGTTTGGAGTCCCTGCCATT, reverse AAGCACTGCCTTCTTACTCCG; *Nuclear Factor (Erythroid-Derived 2)-Like 2 (NRF2)*, forward GTGGATCTGCCAACTACTCCC, reverse TACAAACGGGAATGTCTGCG; *Heme Oxygenase 1 (HMOX1)*, forward AGTCTTCGCCCCTGTCTACT, reverse CTTCACATAGCGCTGCATGG; *Glutamate—cysteine ligase catalytic subunit (GCLC)*, forward CGGAGGAACAATGTCCGAGT, reverse TGTTAAGGTACTGGGAAATGAAGT; *Glutamate-cysteine ligase regulatory subunit* (*GCLM)*, forward AGACGGGGAACCTGCTGAA, reverse ATGAAGCTCCTCGCTGTGC; *Thioredoxin reductase 1 (TXNRD1)*, forward ATCATCATTGGAGGTGGCTCA, reverse ACACATGTTCCTCCGAGACC; *Kelch-like ECH-associated protein 1 (KEAP1),* forward TCGATGGCCACATCTATGCC, reverse CCGATCCTTCGTGTCAGCAT.

### 2.4. Immunoprecipitation

Cells were lysed with IP lysis buffer and equal concentrations of protein incubated with 5 µL of p62 antibody (Abnova, Walnut, CA, USA; L2011-2C11) or ms IgG (Thermo Scientific, Invitrogen, Waltham, MA, USA; 31903) or 5 µL of KEAP1 antibody (Cell Signaling, Danvers, MA, USA, 4678S) or rabbit IgG (Cell Signaling, Danvers, MA, USA, 2729S). Immunoprecipitation (IP) was performed using Pierce Magnetic co-IP kit (Thermo Scientific; 88804) per manufacturer’s instructions.

### 2.5. Protein Identification and Relative Quantification by Gel LCMS

Proteins in the eluates were separated by 1-D SDS-PAGE using a Criterion XT 12% gel that was electrophoresed for 1.5 cm and then stained with Coomassie blue. The protein-containing region of each gel lane was divided into six slices that were individually reduced in situ with TCEP [tris(2-carboxyethyl)phosphine] and alkylated in the dark with iodoacetamide prior to treatment with trypsin (Promega, Madison, WI, USA). The digests were analyzed by capillary HPLC-electrospray ionization tandem mass spectrometry on a Thermo Fisher LTQ Orbitrap Velos Pro mass spectrometer fitted with a New Objective Digital PicoView 550 NanoESI source. On-line HPLC separation was accomplished with an RSLC NANO HPLC system (Thermo Scientific/Dionex, Waltham, MA, USA): [column, PicoFrit™ (New Objective; 75 μm i.d.) packed to 15 cm with C18 adsorbent (Avantor/Vydac, Allentown, PA, USA; 218MS 5 μm, 300 Å)]. Precursor ions were acquired in the orbitrap from *m*/*z* 300–*m*/*z* 2000 in profile mode at 60,000 resolution (*m*/*z* 400); data-dependent acquisition collision-induced dissociation spectra of the six most intense ions in each precursor scan were acquired at the same time in the linear trap (MS2 isolation width, 3; unassigned charge states rejected; normalized collision energy was 30; dynamic exclusion repeat count was 1; and dynamic exclusion repeat duration was 30 s). Mascot (v2.6.2; Matrix Science) was used to search the spectra against a combination of the following databases: UniProt_Human_20181204 (95,936 sequences, 38,067,061 residues); antibodies used for pull-down experiments (90 sequences; 16,712 residues); and common contaminants (247 sequences; 128,130 residues). Cysteine carbamidomethylation was set as a fixed modification and methionine oxidation, and deamidation of glutamine and asparagine were considered as variable modifications; trypsin was specified as the proteolytic enzyme, with two missed cleavages allowed. The Mascot results files for the six gel slices in each lane were combined in Scaffold (v4.9.0; Proteome Software, Portland, OR, USA) for subset searching of the identified proteins by X! Tandem, cross-correlation with the Mascot results, and determination of protein and peptide identity probabilities. The thresholds for acceptance of peptide and protein assignments in Scaffold were 95% and 99%, respectively, two peptides minimum per protein, resulting in a protein-level FDR of 0.2% [46]. Scaffold (v4.9.0; Proteome Software, Portland, OR, USA) was used for all MS data processing, and the non-normalized exclusive spectrum count was compared.

### 2.6. Protein Identification and Relative Quantification by Data-Independent Acquisition Mass Spectrometry (DIA-MS)

LNCaP, LNas1, and LNbs1 were treated with normal 10% DMEM or serum starved (0% DMEM) for 3 days. Cells were lysed with IP lysis buffer and equal concentrations of protein incubated with 5 µL of p62 antibody (Abnova, Walnut, CA, USA; L2011-2C11), or ms IgG (Thermo Scientific, Invitrogen, Waltham, MA, USA; 31903), or 5 µL of KEAP1 antibody (Cell Signaling, Danvers, MA, USA; 4678S), or rabbit IgG (Cell Signaling, Danvers, MA, USA, 2729S). Immunoprecipitation (IP) was performed using Pierce MS-compatible Magnetic co-IP kit (Thermo Scientific, Waltham, MA, USA; 90409) per manufacturer’s instructions. Aliquots of the pull-down eluates (42 µL) were mixed with a buffer containing 10% SDS/50 mM triethylammonium bicarbonate (TEAB) in the presence of protease and phosphatase inhibitors (Halt; Thermo Scientific, Waltham, MA, USA) and nuclease (Pierce™ Universal Nuclease for Cell Lysis; Thermo Scientific, Waltham, MA, USA), reduced with tris(2-carboxyethyl)phosphine hydrochloride (TCEP), alkylated in the dark with iodoacetamide and applied to S-Traps (micro; Protifi) for tryptic digestion (sequencing grade; Promega, Madison, WI, USA) in 50 mM triethylammonium bicarbonate (TEAB). Peptides were eluted from the S-Traps with 0.2% formic acid in 50% aqueous acetonitrile. Data-independent acquisition mass spectrometry (DIA-MS) was conducted on an Orbitrap Fusion Lumos mass spectrometer (Thermo Scientific, Waltham, MA, USA). On-line HPLC separation was accomplished with an RSLC NANO HPLC system (Thermo Scientific/Dionex, Waltham, MA, USA: column, PicoFrit™ (New Objective; 75 μm i.d.) packed to 15 cm with C18 adsorbent (Avantor/Vydac, Allentown, PA, USA; 218MS 5 μm, 300 Å); mobile phase A, 0.5% acetic acid (HAc)/0.005% trifluoroacetic acid (TFA) in water; mobile phase B, 90% acetonitrile/0.5% HAc/0.005% TFA/9.5% water; gradient 3 to 42% B in 120 min; and flow rate, 0.4 μL/min. A pool was made of all digests, and aliquots were analyzed using gas-phase fractionation and 4-*m*/*z* windows (three mass windows, staggered; 30k resolution for precursor and product ion scans, all in the orbitrap) to create an empirically corrected DIA chromatogram library [47] by searching against a Prosit-generated predicted spectral library [48] based on a combination of the following databases: UniProt_Human_20191022 (20,365 sequences; 11,359,329), bovine serum proteins [49] (199 sequences; 145,316 residues), and common contaminants (124 sequences; 62,564 residues). Experimental samples were blocked by replicate and randomized within each replicate for sample preparation and analysis. MS data for experimental samples were acquired in the orbitrap using 8-*m*/*z* windows (staggered; 30k resolution for precursor and product ion scans) and searched against the chromatogram library. Scaffold DIA (v3.2.1; Proteome Software, Portland, OR, USA) was used for all DIA-MS data processing [47,48,49], and non-normalized exclusive intensity was compared. Scaffold DIA exclusive intensity is defined as the summarized intensity value of only the peptides that are associated with this protein group and no other.

### 2.7. Western Blot

Protein isolation and western blot were performed as previously described in [21]. *Primary antibodies:* p62 (Abnova, Walnut, CA, USA; L2011-2C11), KEAP1 (Cell Signaling, Danvers, MA, USA; 8074T), p-p62 (Ser349) (Cell Signaling, Danvers, MA, USA; 95697), GCLC (Cell Signaling, Danvers, MA; 48005S), HO-1 (Cell Signaling, Danvers, MA, USA; 43966S), and β-actin (Santa Cruz, Santa Cruz, CA, USA; sc-69879). *Secondary antibodies:* Sheep anti-mouse (Jackson ImmunoResearch Laboratories, Grove, PA, USA; 515-035-062) and goat anti-rabbit (Abnova, Walnut, CA, USA; PAB10822). Western blot densitometry was performed using Image J, version 1.50d (National Institutes of Health, Bethesda, MD, USA). β-actin is the western blot loading control and the protein/β-actin ratio is normalized to treatment control for densitometry.

### 2.8. Statistical Analysis

Statistical significance for RNA was determined using an unpaired Student *t*-test calculated using Microsoft Excel. *p*-values of ≤0.05 were considered to be statistically significant and denoted by asterisks (* *p* ≤ 0.05; ** *p* ≤ 0.005; *** *p* ≤ 0.0005). Graphs are shown as the average of a minimum of n = 3 biological replicates +/− standard deviation (STDEV).

## 3. Results

### 3.1. The p62-KEAP1 Complex Accumulation Is Basally High in Chronic IL-1 Sublines

**Figure 1:**
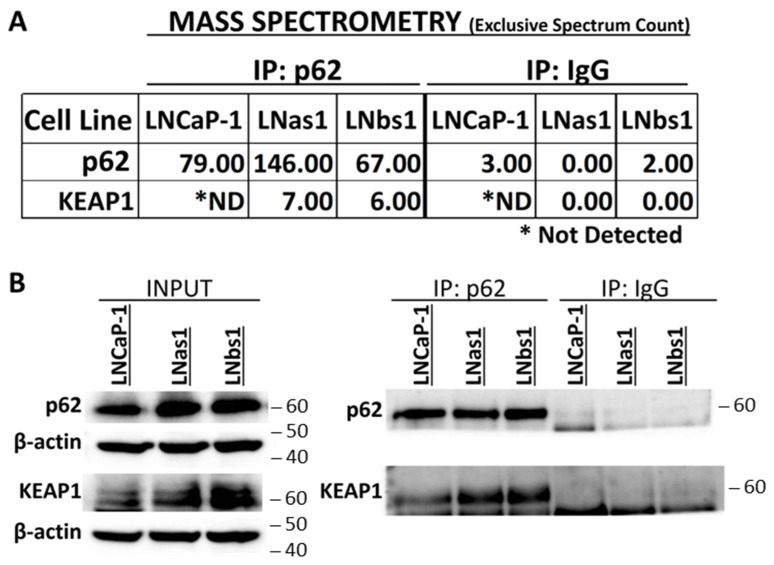
Chronic IL-1 sublines have basally high accumulation of the p62-KEAP1 interaction. (**A**) Mass spectrometry using gel LCMS was performed for p62 immunoprecipitates from LNCaP-1, LNas1, and LNbs1 cells grown in normal growth medium containing 10% serum. Mass spectrometry was analyzed using Scaffold software. Values shown are Scaffold “exclusive spectrum count”. LNas1 and LNbs1 show high basal accumulation of the p62-KEAP1 interaction in 10% serum, whereas no KEAP1 interaction is detected in LNCaP-1 parentals. (**B**) p62 immunoprecipitation (IP) was performed for LNCaP-1, LNas1, and LNbs1 cells grown in normal growth medium containing 10% serum, followed by western blot for p62 and KEAP1. LNas1 and LNbs1 show high basal accumulation of the p62-KEAP1 interaction. IgG IP is the negative control for p62 IP. ND = not detected.

Canonical IL-1/NFκB signaling is mediated through p62-TRAF6 binding [37,50]. We previously reported that the chronic IL-1 sublines, LNas1 and LNbs1, are insensitive to exogenous IL-1 inflammatory signaling [21], and therefore chronic IL-1 sublines likely do not have a competent IL-1/NFκB signaling pathway mediated by p62-TRAF6. Thus, to begin to understand the role of p62 in the chronic IL-1 sublines, we performed mass spectrometry to identify p62 binding partners. We performed Gel LCMS on the LNCaP parental (LNCaP-1) cell line and the LNas1 and LNbs1 chronic IL-1 sublines grown in normal growth medium containing 10% serum. Gel LCMS detected KEAP1 protein in the p62 immunoprecipitate from the subline cells, but KEAP1 protein was not detected in the parental cell line (Figure 1A). We confirmed the mass spectrometry result by performing p62 immunoprecipitation followed by western blot for p62 and KEAP1 (Figure 1B) and in two additional sets of chronic IL-1 sublines that we generated (LNas2, LNbs2, LNas3, LNbs3; Figure S2). Thus, chronic IL-1 exposure reproducibly selects for cells that acquire high basal accumulation of the p62-KEAP1 complex.

### 3.2. Chronic IL-1 Sublines Have High Basal NRF2 Target Genes, GCLC, and HMOX1

**Figure 2:**
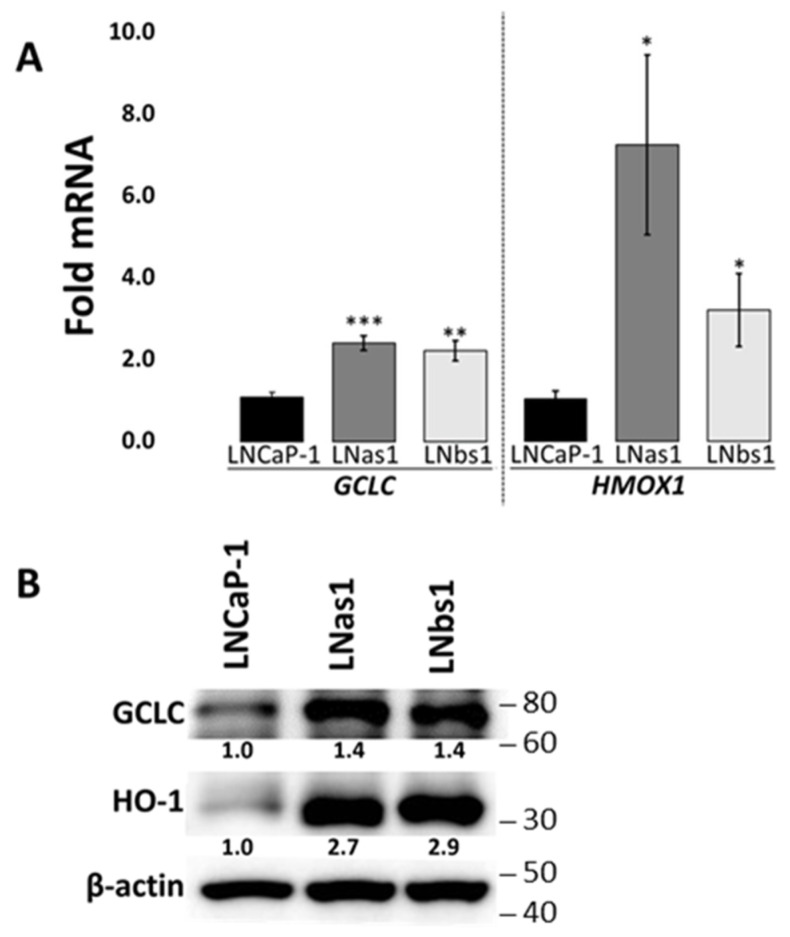
Chronic IL-1 sublines express high basal levels of the NRF2 target genes, *GCLC,* and *HMOX1*. LNCaP-1, LNas1, and LNbs1 were grown in normal growth medium containing 10% serum for 3 days and mRNA and protein isolated. (**A**) RT-qPCR and (**B**) western blot show that GCLC and HMOX1 are basally high in LNas1 and LNbs1 as compared to LNCaP-1 parental cells. Protein densitometry normalized to β-actin loading control and LNCaP-1. mRNA levels are normalized to LNCaP-1. Error bars, ±STDEV of 3 biological replicates; *p*-value, * ≤ 0.05, ** ≤ 0.005, *** ≤ 0.0005.

Because p62 binds and sequesters KEAP1 to enable NRF2 nuclear translocation and transactivation [51,52,53], we hypothesized that the high basal accumulation of the p62-KEAP1 interaction observed in the chronic IL-1 sublines promotes constitutive NRF2 activity. Therefore, to assess basal NRF2 activity we performed RT-qPCR for canonical NRF2 target genes *GCLC*, *GCLM*, *HMOX1*, *NQO1*, and *TXNRD1* in the LNCaP parental cells and chronic IL-1 subline cells under normal growth conditions. In line with our previously reported RNA sequencing data for LNCaP-1 (parental cell line), LNas1, and LNbs1 [22], we did not detect high basal levels of the NRF2 target genes *GCLM*, *NQO1*, or *TXNRD1* conserved among the chronic IL-1 sublines (i.e., LNas1, LNas2, LNas3, LNbs1, LNbs2, LNbs3) (Figure S3). However, we did detect high basal levels of *GCLC* and *HMOX1* mRNA in all three sets of chronic IL-1 sublines (Figure 2 and Figure S4). These data suggest that while overall NRF2 activity is not basally high in the subline cells, chronic IL-1 exposure reproducibly leads to constitutive transactivation of a subset of NRF2 target genes.

### 3.3. NRF2 Is Basally Active in LNCaP Parental and Chronic IL-1 Subline Cells

**Figure 3:**
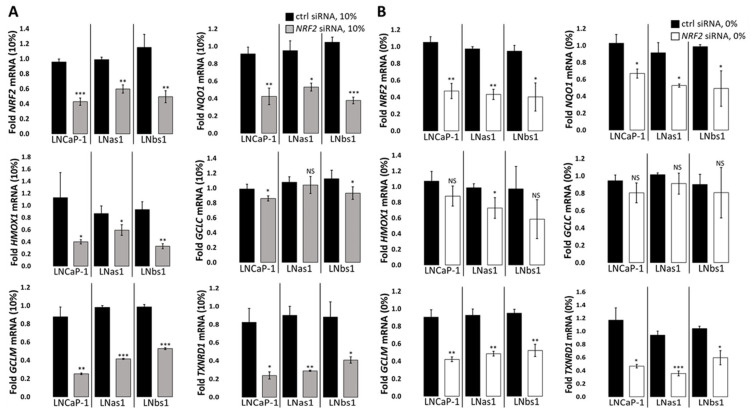
NRF2-dependent target gene expression in LNCaP-1, LNas1, and LNbs1. *NRF2* was siRNA silenced in LNCaP-1, LNas1, and LNbs1 cells grown in normal growth medium containing 10% serum or 0% serum for 4 days and mRNA and protein isolated. RT-qPCR shows that NRF2 siRNA attenuates canonical NRF2-induced target gene expression in LNCaP-1, LNas1, and LNbs1 under (**A**) serum replete (10%) and/or (**B**) serum starvation (0%) for *NQO1*, *HMOX1*, *GCLM*, and *TXNRD1*, but not *GCLC*. mRNA levels are normalized to (**A**) 10% serum or (**B**) 0% serum, ctrl siRNA within each cell line. Error bars, ±STDEV of 3 biological replicates; *p*-value, * ≤ 0.05, ** ≤ 0.005, *** ≤ 0.0005; NS = not significant.

While RNA sequencing [21] and RT-qPCR for canonical genes (Figure S3) suggest that overall NRF2 activity is not elevated in the chronic IL-1 subline cells, we determined if NRF2 is basally active in LNCaP parental and chronic IL-1 subline cells. To determine if NRF2 is basally active in LNCaP parental and chronic IL-1 subline cells, cells were grown in normal growth medium supplemented with 10% serum and transfected with *NRF2* siRNA. RT-qPCR shows that *NRF2* siRNA was sufficient to reduce mRNA levels of *NRF2* and NRF2 target genes, *NQO1, GCLM, HMOX1,* and *TXNRD1* mRNA in LNCaP-1, LNas1, and LNbs1 cells (Figure 3A), indicating that NRF2 is basally active. Notably, mRNA levels of the NRF2 target gene, *GCLC*, were not significantly reduced (Figure 3A), suggesting that NRF2 does not induce *GCLC* expression under basal growth conditions in the LNCaP cell line background. In addition, these results indicate that chronic IL-1 exposure does not alter basal NRF2 activity in the LNCaP cell line background.

### 3.4. Chronic IL-1 Sublines Show Hypersensitive Regulation of Glutamate-Cysteine Ligase Catalytic Subunit (GCLC) and Heme Oxygenase 1 (HMOX1/HO-1) in Response to Serum Starvation

**Figure 4:**
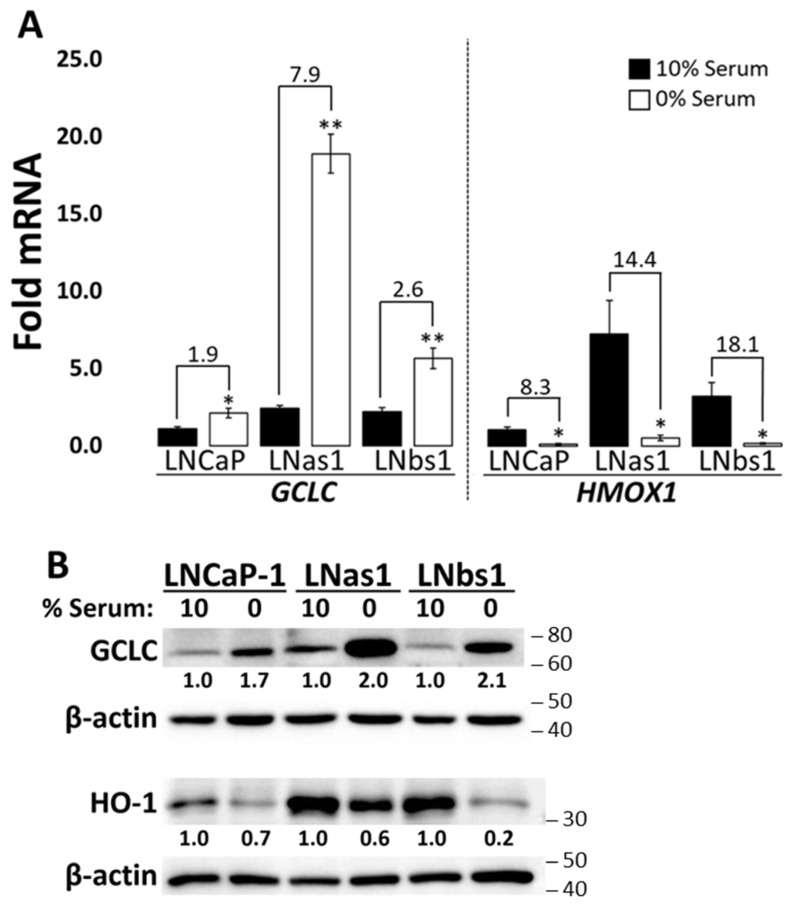
Chronic IL-1 sublines are hypersensitive to serum starvation regulation of GCLC and HMOX1 levels. LNCaP-1, LNas1, and LNbs1 were grown in normal growth medium containing 10% serum or 0% serum for 3–4 days and mRNA and protein isolated. (**A**) RT-qPCR and/or (**B**) western blot show that GCLC and HMOX1 are induced or repressed, respectively, in the absence of serum in LNCaP-1, LNas1, and LNbs1. LNas1 and LNbs1 are hypersensitive to serum starvation. mRNA levels are normalized to LNCaP-1, 10% serum, and fold change values are indicated. Error bars, ±STDEV of 3 biological replicates; *p*-value, * ≤ 0.05, ** ≤ 0.005; NS = not significant. Protein densitometry normalized to β-actin loading control and 10% serum.

As stated above, chronic IL-1 sublines have high basal levels of GCLC and HMOX1 (Figure 2), and nutrient starvation has been reported to regulate *GCLC* [54] and *HMOX1* [55] expression, whereas cysteine starvation induces *GCLC* expression and serum starvation reduces *HMOX1* expression. Therefore, we assessed GCLC and HMOX1 mRNA levels and protein accumulation under serum starvation conditions in LNCaP-1, LNas1, and LNbs1. Cells were grown in normal growth medium in the presence (10%) or absence (0%) of serum. Serum starvation increased *GCLC* mRNA levels 1.9-fold, 7.9-fold, or 2.6-fold in LNCaP-1, LNas1, or LNbs1, respectively (Figure 4A) and reduced *HMOX1* levels 8.3-fold, 14.4-fold, or 18.1-fold (Figure 4A). Similar results were observed for GCLC and HMOX1 protein accumulation (Figure 4B). Thus, the chronic IL-1 sublines, LNas1 and LNbs1 are hypersensitive to serum starvation-mediated regulation of GCLC and HMOX1 levels. Similar results were observed for the other chronic IL-1 sublines (i.e., LNas2, LNas3, LNbs2, LNbs3) (Figure S5). Together these data suggest that chronic IL-1 exposure selects for cells that evolve hypersensitivity to serum starvation-mediated regulation of *GCLC* and *HMOX1* expression.

### 3.5. Serum Starvation Induces p62-KEAP1 Complex Accumulation in LNCaP Cells

**Figure 5:**
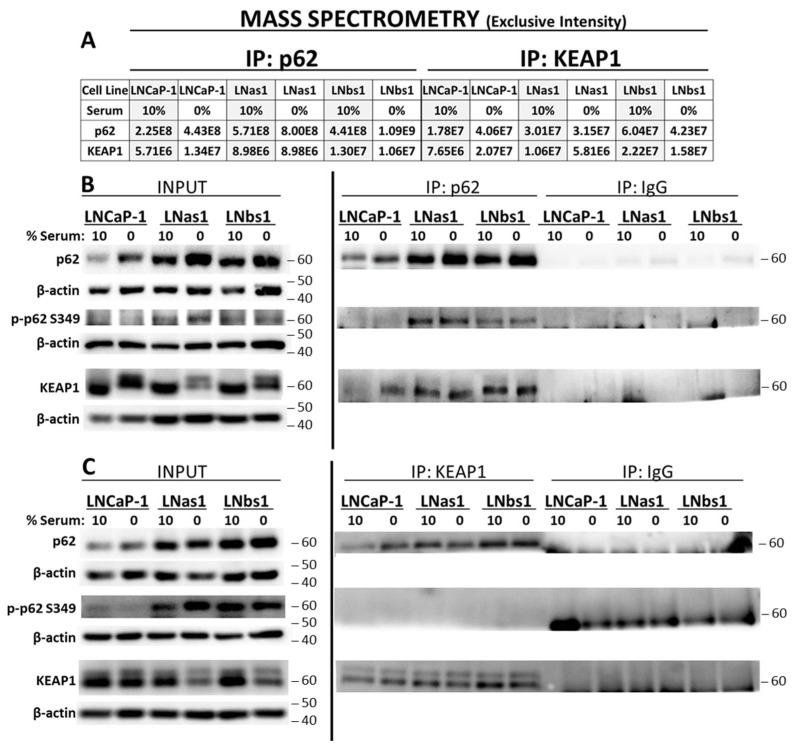
The p62-KEAP1 complex accumulation is upregulated by serum starvation in LNCaP parental cells and constitutive in subline cells. (**A**) DIA mass spectrometry was performed for LNCaP-1, LNas1, and LNbs1 cells grown in normal growth medium containing 10% serum or 0% serum for 2 days. p62 or KEAP1 were immunoprecipitated (IP) from LNCaP-1, LNas1, or LNbs1 cells. DIA mass spectrometry was analyzed using Scaffold DIA software. Values shown are non-normalized exclusive intensity. As compared to 10% serum, p62 and KEAP1 exclusive intensity are greater in 0% serum in LNCaP-1 parental cells for both p62 IP and KEAP1 IP, suggesting that 0% serum starvation induces accumulation of the p62-KEAP1 complex in LNCaP-1 cells. An increase in exclusive intensity in 0% versus 10% serum is not observed for LNas1 or LNbs1, suggesting that serum starvation does not induce p62-KEAP1 complex formation in the sublines. (**B**,**C**) IP followed by western blot was performed for LNCaP-1, LNas1, and LNbs1 cells grown in normal growth medium containing 10% serum or 0% serum for 2 days, and blots were probed for p62, p-p62 (SER349), or KEAP1. (**B**) p62 or (**C**) KEAP1 were IP’d from LNCaP-1, LNas1, or LNbs1 cells. Input western blots show that 0% serum induces p62 accumulation in LNCaP-1 parental cells, and IP western blots show that 0% induces p62-KEAP1 interaction in LNCaP-1 parental cells. Input and IP western blots show that in comparison to parental cells, p62 levels and p62-KEAP1 complex formation are basally higher in the sublines (e.g., 10% serum). In addition, input and IP western blots show that, in comparison to 10% serum, 0% serum reduces KEAP1 accumulation in the sublines, yet the p62-KEAP1 complex formation is comparable to parental 0% and subline 10%, suggesting the complex is constitutive and stable. Finally, p-p62 (SER349) is found only in the subline p62 immunoprecipitates. Thus, KEAP1 does not bind p-p62 (SER349) in the LNCaP-1 parental or subline cells grown in 10% or 0% serum. β-actin is the western blot loading control. IgG IP is the negative control.

Given that the chronic IL-1 sublines are hypersensitive to starvation-induced regulation of *GCLC* and *HMOX1* expression, two canonical NRF2 genes, we wanted to determine if serum starvation, in kind, altered the p62-KEAP1 interaction. We performed p62 or KEAP1 IP, followed by DIA mass spectrometry, for LNCaP-1, LNas1, and LNbs1 cells grown in the presence (10%) or absence (0%) of serum. Shown are the peptide exclusive intensities for p62 and KEAP1 (Figure 5A). Mass spectrometry of LNCaP parental cells reveals that, for both the p62 IP and KEAP1 IP, the p62 and KEAP1 peptide exclusive intensities are greater in 0% serum than in 10% serum, indicating that serum starvation induces accumulation of the p62-KEAP1 complex in LNCaP cells (Figure 5A). We confirmed this result with IP western (Figure 5B,C).

Mass spectrometry (Figure 5A) and IP western blot (Figure 5B,C) of LNas1 and LNbs1 cells show that serum starvation does not increase p62-KEAP1 complex accumulation over serum replete conditions in the subline cells. Of note, serum starvation reduces KEAP1 levels in the subline cells (Figure 5A, KEAP1 IP; Figure 5B,C, input), yet the relative levels of p62 that immunoprecipitate with KEAP1 in the KEAP1 IP remain comparable in 10% versus 0% serum (Figure 5A, KEAP1 IP; Figure 5C, KEAP1 IP). This suggests that the p62-KEAP1 interaction in the subline cells is constitutive and stable.

Finally, we investigated if phosphorylated p62 ^SER349^ was bound to KEAP1 in parental or subline cells. p62^SER349^ phosphorylation facilitates p62 binding to KEAP1 but is not required for binding [56]. We detect p62^SER349^ phosphorylation in the subline p62 IP for 10% and 0% serum (Figure 5B,C). However, parental cells do not appear to accumulate phosphorylated p62^SER349^ in response to serum starvation, nor does KEAP1 appear to bind phosphorylated p62 ^SER349^ in the LNCaP parental or subline cells. This suggests that p62 ^SER349^ does not regulate p62-KEAP1 interaction in subline cells or in response to serum starvation in parental or subline cells.

Taken together, our mass spectrometry and IP western results show that p62 and KEAP1 interact under serum starvation conditions in both parental and subline cells. Therefore, we speculated that the p62-KEAP1 interaction may regulate NRF2-dependent *GCLC* and *HMOX1* expression in response to nutrient stress.

### 3.6. GCLC Expression Is NRF2/KEAP1/p62-Independent in the LNCaP Background

*GCLC* expression is both basally high (Figure 2 and Figure S4) and strongly induced by serum starvation (Figure 4 and Figure S5) in the chronic IL-1 sublines, and while NRF2 did not regulate *GCLC* expression under serum replete growth conditions in parental nor the chronic IL-1 sublines cells (Figure 3A), we wanted to determine if the NRF2/KEAP1/p62 axis mediated the hypersensitive induction of *GCLC* expression in serum-starved chronic IL-1 sublines. To determine if *GCLC* expression is upregulated by the NRF2/KEAP1/p62 axis in response to serum starvation, we siRNA silenced *NRF2*, *KEAP1*, or *p62* in LNCaP-1, LNas1, and LNbs1 cells grown in the absence of serum. As expected, RT-qPCR for the canonical NRF2 target genes showed downregulation of *NQO1*, *GCLM*, and *TXNRD1* (Figure 3B) in serum-starved LNCaP-1, LNas1, and LNbs1. Thus, NRF2 is active under serum starvation conditions. Unexpectedly, *NRF2* siRNA did not reduce GCLC parental nor the chronic IL-1 cells under serum starvation conditions (Figure 3B). This suggests the hypersensitive induction of *GCLC* expression in serum-starved chronic IL-1 sublines is NRF2-independent.

**Figure 6:**
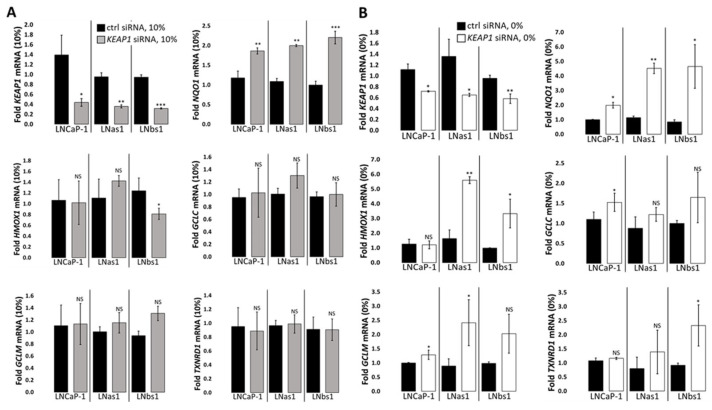
KEAP1-dependent target gene expression in LNCaP-1, LNas1, and LNbs1. *KEAP1* was siRNA silenced in LNCaP-1, LNas1, and LNbs1 cells grown in normal growth medium containing 10% serum or 0% serum for 4 days, and mRNA was isolated for RT-qPCR. RT-qPCR shows that *KEAP1* silencing is sufficient to upregulate NRF2 target genes, *NQO1*, *HMOX1*, *GCLM*, and *TXNRD1* but not *GCLC* in (**A**) serum replete (10%) and/or (**B**) serum starvation (0%) conditions in both parental and chronic IL-1 subline cells. mRNA levels are normalized to (**A**) 10% serum or (**B**) 0% serum, ctrl siRNA within each cell line. Error bars, ±STDEV of 3 biological replicates; *p*-value, * ≤ 0.05, ** ≤ 0.005, *** ≤ 0.0005; NS = not significant.

KEAP1 is a negative regulator of NRF2 activity [52,57]. Using *NQO1* expression as a surrogate for canonical NRF2 activity, as expected, *KEAP1* siRNA upregulated *NQO1* expression under serum replete (Figure 6A) and deplete (Figure 6B) conditions in parental and chronic IL-1 subline cells. However, similar to *NRF2* siRNA (Figure 3), *KEAP1* siRNA had no effect on *GCLC* expression under serum replete (Figure 6A) or deplete (Figure 6B) conditions in parental and chronic IL-1 subline cells. These results imply that the hypersensitive induction of *GCLC* expression in serum-starved chronic IL-1 sublines is KEAP1-independent.

**Figure 7:**
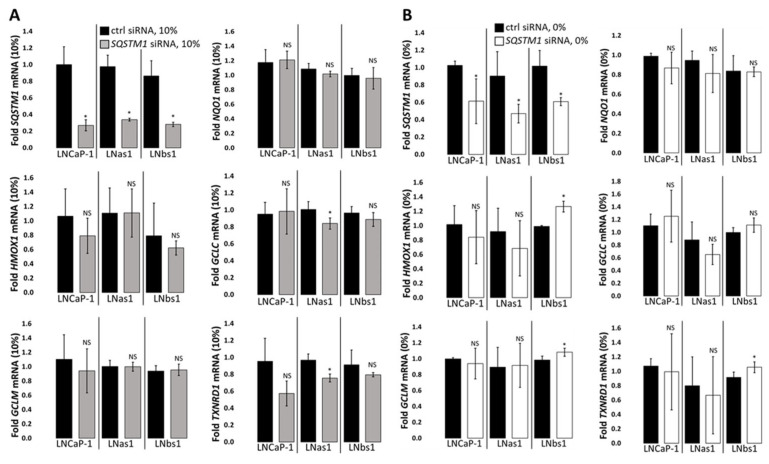
p62-dependent target gene expression in LNCaP-1, LNas1, and LNbs1. *p62* was siRNA silenced in LNCaP-1, LNas1, and LNbs1 cells grown in normal growth medium containing 10% serum or 0% serum for 4 days, and mRNA was isolated for RT-qPCR. RT-qPCR shows that *p62* silencing is not sufficient to repress NRF2 target genes, *NQO1*, *HMOX1*, *GCLC, GCLM*, or *TXNRD1* in (**A**) serum replete (10%) or (**B**) serum starvation (0%) conditions in parental and chronic IL-1 subline cells. mRNA levels are normalized to (**A**) 10% serum or (**B**) 0% serum, ctrl siRNA within each cell line. Error bars, ±STDEV of 3 biological replicates; *p*-value, * ≤ 0.05; NS = not significant.

p62 promotes NRF2 activity by sequestering KEAP1 from NRF2 [51,52,53]. Unexpectedly, however, *p62* siRNA had no effect on *NQO1* expression under serum replete (Figure 7A) or deplete (Figure 7B) conditions in parental and chronic IL-1 subline cells. In kind, *p62* siRNA had no effect on *GCLC* expression in the presence (Figure 7A) or absence (Figure 7B) of serum. Therefore, the hypersensitive induction of *GCLC* expression in serum-starved chronic IL-1 sublines appears to be p62-independent. Taken together, basal and serum starvation-induced *GCLC* expression is NRF2/KEAP1/p62 axis-independent in the LNCaP background, including in the chronic IL-1 sublines.

### 3.7. NRF2/KEAP1 Regulates HMOX1 Expression Independently of p62

While *HMOX1* expression is basally high in chronic IL-1 sublines under normal growth conditions (Figure 2 and Figure S4), the chronic IL-1 sublines are hypersensitive to serum starvation repression of *HMOX1* expression (Figure 4 and Figure S5). Given that NRF2 induces *HMOX1* expression levels in LNCaP-1, LNas1, and LNbs1 under serum replete growth conditions (Figure 3A), we wanted to determine if the NRF2/KEAP1/p62 axis played a role in starvation-mediated *HMOX1* repression. We siRNA silenced *NRF2*, *KEAP1*, or *p62* in LNCaP-1, LNas1, and LNbs1 cells grown in the presence or absence of serum. As stated earlier, under serum replete conditions, *NRF2* siRNA was sufficient to downregulate *HMOX1* expression in the presence of serum (Figure 3A), but because *HMOX1* is highly repressed by serum starvation, *NRF2* siRNA was not sufficient to further repress *HMOX1* in serum-starved LNCaP-1, LNas1, or LNbs1 cells (Figure 3B). As expected, *KEAP1* siRNA was sufficient to increase *HMOX1* levels in LNas1 and LNbs1 under serum starvation conditions (Figure 6B) but was not sufficient to increase the already high basal (serum replete) *HMOX1* levels in the chronic IL-1 sublines (Figure 6A). Notably, *KEAP1* siRNA has no effect on *HMOX1* levels in LNCaP-1 cells under serum replete (Figure 6A) or deplete (Figure 6B) conditions. Finally, as observed for *NQO1* and *GCLC*, *p62* siRNA was not sufficient to reduce *HMOX1* levels in LNCaP-1, LNas1, or LNbs1 under serum replete (Figure 7A) or deplete (Figure 7B) conditions. Together, these data imply that the NRF2/KEAP1 axis regulates *HMOX1* levels in the chronic IL-1 sublines independent of p62.

## 4. Discussion

We previously found that chronic IL-1 exposure selects for PCa cells that evolve resistance to IL-1-induced cytotoxicity and cytostaticity [21,22]. Our data show that PCa cells can evolve resistance to the anti-tumorigenic effects of acute inflammation and, in turn, co-opt the acquired resistance mechanism(s) to mitigate cellular stress caused by other stimuli, such as nutrient deprivation. One consequence of nutrient deprivation (i.e., serum starvation) is attenuated AR activity [21,58], and we found that LNCaP cells chronically exposed to IL-1 evolve AR independence, rendering the cells insensitive to the cytotoxic effects of AR loss [21]. Because p62 mediates acute IL-1 signaling [59] and p62 promotes cell survival of AR-independent PCa cells [13,15], we sought to determine the function of p62 in LNCaP cells chronically exposed to IL-1.

p62 is a multi-functional prosurvival protein that has many different binding partners, each reflecting distinct regulation and roles for p62 [51,53]. To begin to uncover p62 regulation and function in PCa cells chronically exposed to IL-1, we performed mass spectrometry to identify p62 binding proteins both in the presence and absence of serum in LNCaP parental and chronic IL-1 subline cells. Mass spectrometry, as confirmed by IP western blot, revealed high basal accumulation of the p62-KEAP1 complex in the chronic IL-1 sublines under serum replete conditions (Figure 1, Figure 5 and Figure S2). Serum starvation reduced KEAP1 levels in the sublines resulting in reduced levels of KEAP1 immunoprecipitate in the KEAP1 IP. Notably, however, the relative amount of p62 that immunoprecipitated with KEAP1 in the KEAP1 IP was still comparable to serum replete conditions (Figure 5A,C), suggesting that the p62-KEAP1 complex formation in the chronic IL-1 sublines is constitutive and stable.

Serum starvation induced p62-KEAP1 interaction in LNCaP parental cells (Figure 5), and because serum starvation induces autophagy [60,61], we expected to find the LC3-p62-KEAP1 complex in serum-starved LNCaP parental and chronic IL-1 subline cells, which would indicate non-canonical activation of NRF2 signaling. Non-canonical NRF2 activation occurs when p62 binds and localizes KEAP1 to the LC3-lipidated autophagosome for degradation [51,52] and p62^SER349^ phosphorylation (p-p62^SER349^) facilitates p62 binding to KEAP1 [56]. However, using mass spectrometry and IP western blot, we were not able to detect p62 nor KEAP1 binding to LC3 in LNCaP parental or chronic IL-1 subline cells in the presence or absence of serum, nor did we detect p-p62^SER349^ bound to KEAP1 using IP western blot (Figure 5C). Notably, SER349 phosphorylation increases binding affinity but is not required for p62 binding to KEAP1 [52] and, thus, may explain why we were unable to detect KEAP1 binding to p-p62^SER349^ in parental or chronic IL-1 subline cells (Figure 5C). Furthermore, using several canonical NRF2 target genes as surrogates for general NRF2 activity, we found that the target genes were regulated independently of p62 in the parental and subline cells (Figure 7) and that overall NRF2 activity was not basally high in the chronic IL-1 sublines cells (Figure S3) despite high basal accumulation of the p62-KEAP1 interaction (Figure 1 and Figure 5). Taken together, the p62-KEAP1 interaction induced by serum starvation in LNCaP parental cells and basally high in the chronic IL-1 sublines does not appear to reflect KEAP1 sequestration into the autophagosome for degradation or NRF2 activation but instead may reflect an altered NRF2-independent p62-KEAP1 regulation and function.

**Figure 8:**
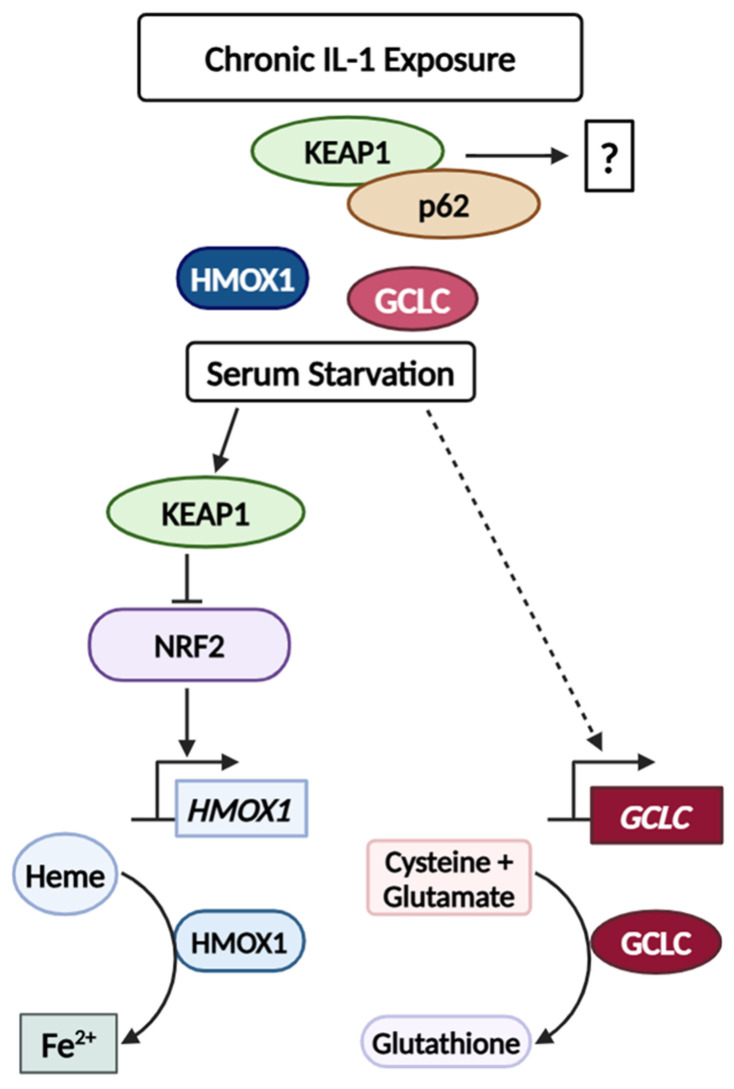
Model. Chronic IL-1 exposure selects for cells that evolve high basal accumulation of NRF2-indepedent p62-KEAP1 interaction, the function of which remains unknown. Chronic IL-1 exposure also selects for cells that have high basal *HMOX1* and *GCLC* and are hypersensitive to serum starvation-induced NRF2-KEAP1-dependent *HMOX1* repression and NRF2-KEAP1-p62-independent *GCLC* upregulation. We speculate that under serum starvation conditions, hypersensitive *HMOX1* repression and *GCLC* induction may protect the chronic IL-1 sublines from ferroptosis by reducing Fe^2+^ and increasing glutathione production, respectively.

Two NRF2 genes that are basally high in the chronic IL-1 sublines are the *glutamate-cysteine ligase catalytic subunit* (*GCLC*) and *heme oxygenase 1* (*HMOX1*/*HO-1*) (Figure 2). Elevated GCLC [62] and HMOX1 [63,64,65] correlate with poor prognosis, tumor progression, and treatment resistance, including CRPC [66]. Thus, high basal GCLC and HMOX1 may support the tumorigenic behavior of cancer cells chronically exposed to IL-1. GCLC is a rate-limiting enzyme that catalyzes the ligation of glutamate and cysteine into glutathione (GSH), a potent antioxidant and inhibitor of ferroptosis (cell death due to the accumulation of ferrous iron (Fe^2+^)) [54,67,68]. In response to nutrient starvation via L-glutamine or cysteine deprivation, NRF2 upregulates GCLC to prevent ferroptosis [54,67]. HMOX1 metabolizes heme into biliverdin, carbon monoxide, and ferrous iron [65,68,69]. Moderately elevated levels of HMOX1 are cytoprotective and will neutralize ROS, preventing ferroptosis [63,65], while overactivation of HMOX1 causes excess ferrous iron production leading to ROS and ferroptosis [63,65]. Thus, it is interesting to speculate that the chronic IL-1 sublines show hypersensitive *GCLC* induction and *HMOX1* repression in response to serum starvation (Figure 4) to protect against ferroptosis (Figure 8).

## 5. Conclusions

In conclusion, using the LNCaP PCa cell line, we have discovered that chronic IL-1 exposure reproducibly selects for cells that acquire high basal accumulation of the p62-KEAP1 complex that appears to be NRF2-independent. Chronic IL-1 exposure also selects for cells that accumulate high basal levels of canonical NRF2 target genes, *GCLC* and *HMOX1*, but we found that the expression of these genes is regulated independently of p62 and that *GCLC* expression is regulated independently of NRF2-KEAP1. This further suggests an NRF2-independent regulation and role for p62-KEAP1 in cells chronically exposed to IL-1. Finally, we found that cells chronically exposed to IL-1 not only have high basal *GCLC* and *HMOX1* expression but also show hypersensitive regulation of GCLC and HMOX1, which might provide a growth advantage under basal growth conditions and against serum starvation. More experiments are needed to determine the regulation and role of p62-KEAP1, GCLC, and HMOX1 acquired in cells chronically exposed to IL-1.

## Data Availability

RNA-seq datasets mentioned in this study are available at GEO NCBI, accession GSE142706.

## Supplementary Materials

The following supporting information can be downloaded at https://www.mdpi.com/article/10.3390/cells14030192/s1, Figure S1: LNCaP response to chronic IL-1 exposure is reproducible and conserved; Figure S2: Chronic IL-1-induced p62-KEAP1 complex accumulation is reproducible and conserved; Figure S3: Chronic IL-1 exposure does not cause a conserved basal elevation of NRF2 target genes, *NQO1*, *GCLM*, and *TXNRD1*; Figure S4: Chronic IL-1 exposure causes a conserved elevation of NRF2 target genes *GCLC* and *HMOX1*; Figure S5: Chronic IL-1-induced hypersensitive serum starvation regulation of *GCLC* and *HMOX1* is reproducible and conserved.
