# Supplementary material for: Chronic IL-1-Exposed LNCaP Cells Evolve High Basal p62-KEAP1 Complex Accumulation and NRF2/KEAP1-Dependent and -Independent Hypersensitive Nutrient Deprivation Response
Source: Cells. 2025 Jan 28;14(3):192. doi: 10.3390/cells14030192 (PMC11816438; doi:10.3390/cells14030192)
Supplement: Supplementary file 1 [file cells-14-00192-s001.zip › cells-3333641-supplementary.pdf]

cells

MDPI

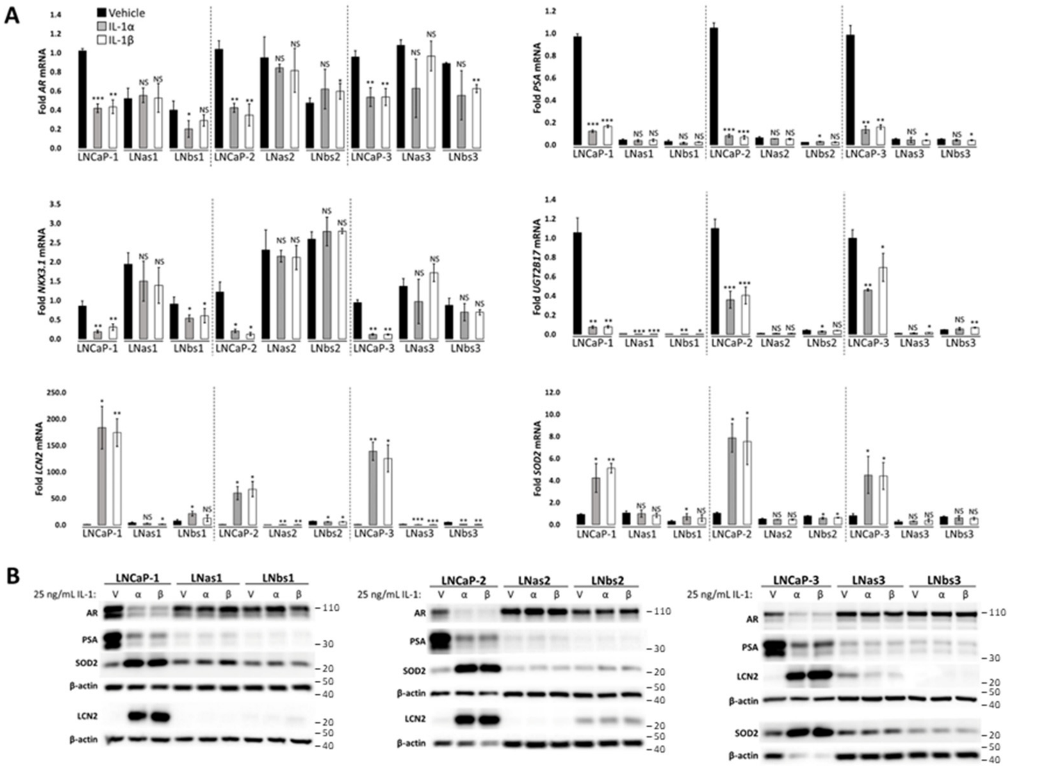

SUPPLEMENTARY FIGURE S1. LNCaP response to chronic IL-1 exposure is reproducible and conserved. We generate three independent sets of LNCaP chronic IL-1 sublines (LNas1, LNbs1, LNas2, LNbs2, LNas3, LNbs3). LNCaP-1, LNas1, and LNbs1 were previously reported in [21]. To confirm response to acute IL-1, LNCaP parental (LNCaP-1, LNCaP-2, LNCaP-3) and the corresponding sublines were treated acutely with vehicle control or 25 ng/mL IL-1α or IL-1β for 2-3 days and mRNA and protein isolated. (A) RT-qPCR and (B) western blot show that AR and AR target genes, *PSA*, *NKX3.1*, and *UGT2B17* are downregulated with IL-1 and IL-1 target genes, *LCN2* and *SOD2*, are upregulated in response to acute IL-1 treatment in the LNCaP parental cells. However, the chronic IL-1 sublines show attenuated or no response to acute IL-1 treatment and show stable repression of PSA. The results indicate that LNCaP response to chronic IL-1 exposure is reproducible and conserved. mRNA levels are normalized to LNCaP parental cells. Error bars, ± STDEV of 3 biological replicates; p-value, *≤0.05, **≤0.005, ***≤0.0005, NS = not significant.

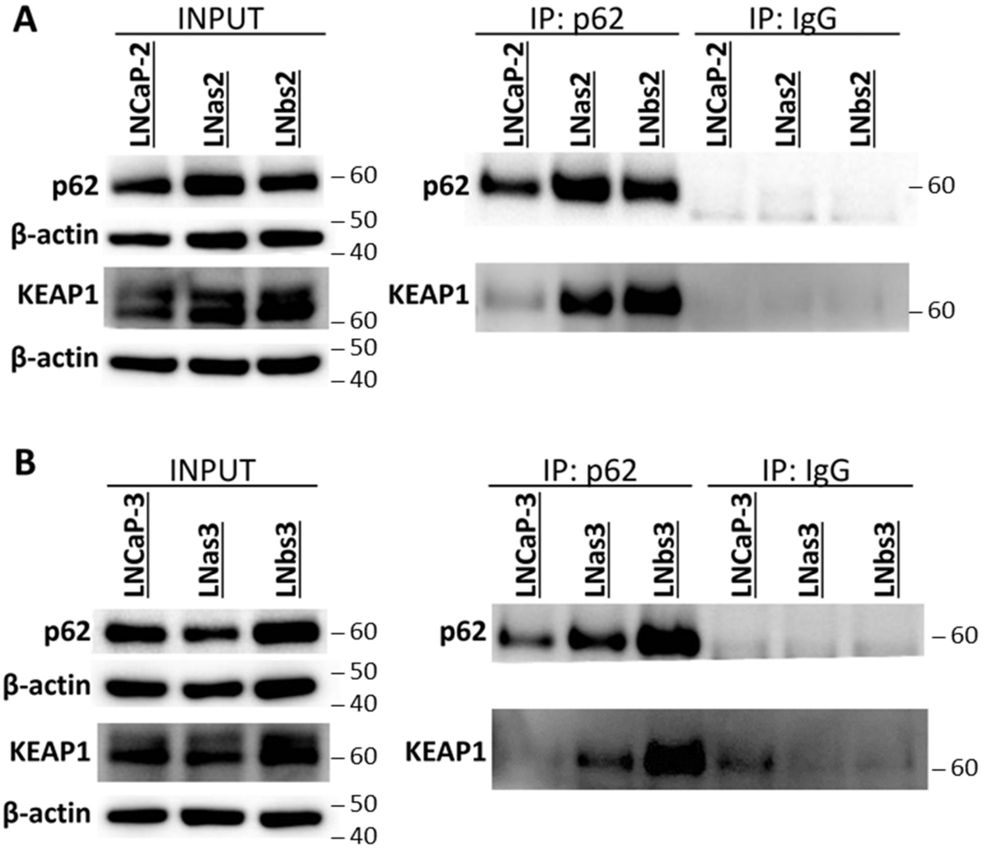

SUPPLEMENTARY FIGURE S2. Chronic IL-1-induced p62-KEAP1 complex accumulation is reproducible and conserved. (A, B) Immunoprecipitation (IP) followed by western blot was performed for LNCaP parental (LNCaP-2, LNCaP-3) and the corresponding chronic IL-1 sublines (LNas2, LNbs2, LNas3, LNbs3) for cells grown in normal growth medium containing 10% serum. Input (left) and IP (right) western blots were probed for p62, KEAP1, or β-actin loading control. IgG IP is the negative control for anti-p62 antibody. IP western blot shows that p62-KEAP1 complex accumulation is basally high in the independent sets of chronic IL-1 sublines and, thus, is conserved and reproducible.

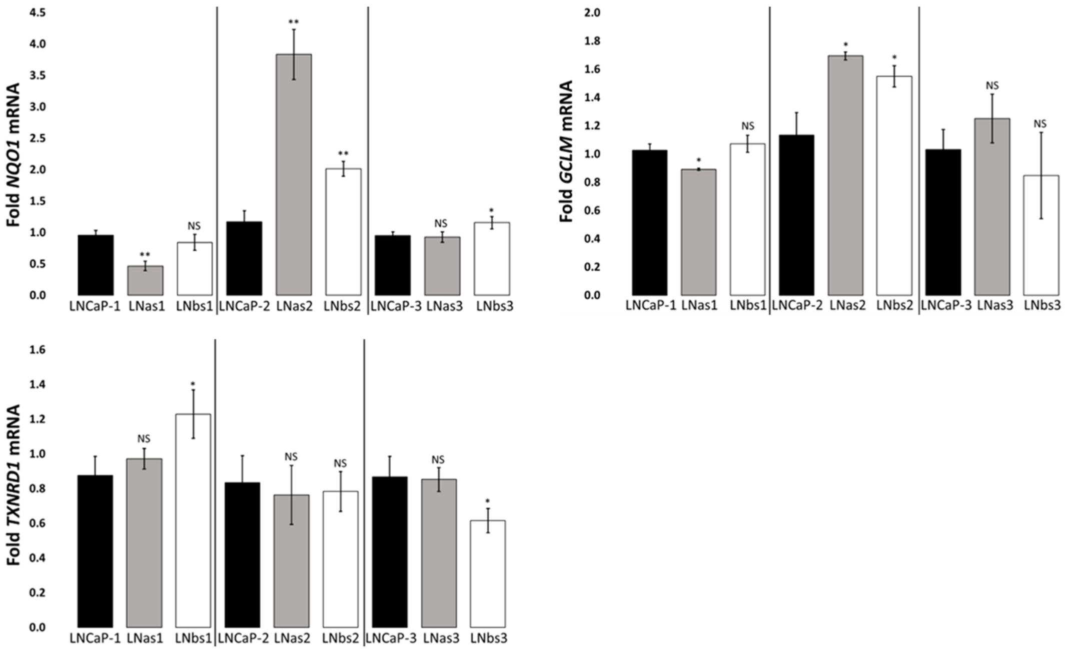

SUPPLEMENTARY FIGURE S3. Chronic IL-1 exposure does not cause a conserved basal elevation of NRF2 target genes, *NQO1*, *GCLM*, and *TXNRD1*. LNCaP parental cells (LNCaP-1, LNCaP-2, LNCaP-3) and the corresponding chronic IL-1 sublines (LNas1, LNbs1, LNas2, LNbs2, LNas3, LNbs3) were grown in normal growth medium containing 10% serum for 3-4 days and mRNA was isolated. RT-qPCR shows that the basal levels of *NQO1*, *GCLM*, and *TXNRD1* mRNA are not consistently elevated across the chronic IL-1 sublines. mRNA levels are normalized to LNCaP parental. Error bars, ± STDEV of 3 biological replicates; p-value, *≤0.05, **≤0.005, NS = not significant.

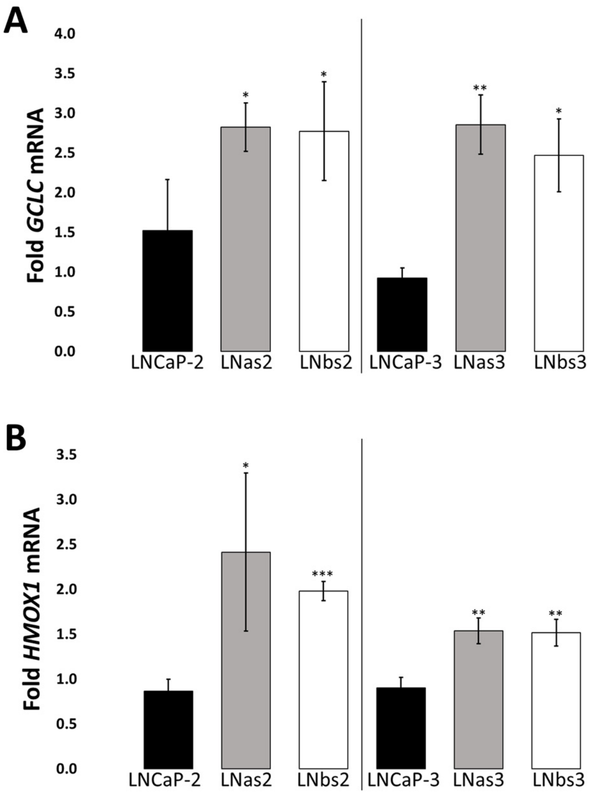

SUPPLEMENTARY FIGURE S4. Chronic IL-1 exposure causes a conserved elevation of NRF2 target genes *GCLC* and *HMOX1*. LNCaP parental (LNCaP-2, LNCaP-3) and the corresponding chronic IL-1 sublines (LNas2, LNbs2, LNas3, LNbs3) were grown in normal growth medium containing 10% serum for 3-4 days and mRNA was isolated. RT-qPCR shows that (A) *GCLC* and (B) *HMOX1* are basally high in the independent sets of chronic IL-1 sublines. Thus, chronic IL-1-induced high basal *GCLC* and *HMOX1* mRNA levels are conserved and reproducible. mRNA levels are normalized to LNCaP parental. Error bars, ± STDEV of 3 biological replicates; p-value, *≤0.05, **≤0.005, ***≤0.0005.

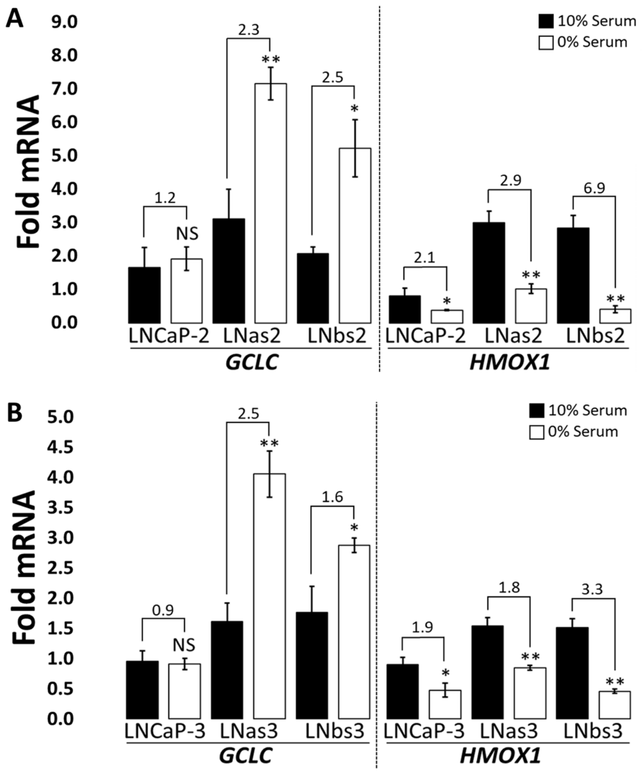

SUPPLEMENTARY FIGURE S5. Chronic IL-1-induced hypersensitive serum starvation regulation of *GCLC* and *HMOX1* is reproducible and conserved. (A, B) LNCaP parental (LNCaP-2, LNCaP-3) and the corresponding chronic IL-1 sublines (LNas2, LNbs2, LNas3, LNbs3) were grown in normal growth medium containing 10% serum or 0% serum for 3-4 days and mRNA isolated. RT-qPCR does not show significant *GCLC* induction in LNCaP parental cells but does show *HMOX1* repression in the absence of serum. The independent sets of chronic IL-1 sublines show hypersensitivity to serum starvation-induced *GCLC* upregulation and *HMOX1* repression. mRNA levels are normalized to LNCaP, 10% serum and fold change values are indicated. Error bars, ±STDEV of 3 biological replicates; p-value, *≤0.05, **≤0.005, NS = not significant.
